# VR09 Cell Line: An EBV-Positive Lymphoblastoid Cell Line with *In Vivo* Characteristics of Diffuse Large B Cell Lymphoma of Activated B-Cell Type

**DOI:** 10.1371/journal.pone.0052811

**Published:** 2012-12-21

**Authors:** Ilaria Nichele, Alberto Zamò, Anna Bertolaso, Francesco Bifari, Martina Tinelli, Marta Franchini, Roberta Stradoni, Fiorenza Aprili, Giovanni Pizzolo, Mauro Krampera

**Affiliations:** 1 Stem Cell Research Laboratory, Section of Hematology, Department of Medicine, University of Verona, Verona, Italy; 2 Section of Pathological Anatomy, Department of Pathology and Diagnostics, University of Verona, Verona, Italy; 3 Laboratory of Cytogenetics, Department of Pathology and Diagnostics, Azienda Ospedaliera Universitaria Integrata Verona, Verona, Italy; University of Navarra, Center for Applied Medical Research, Spain

## Abstract

**Background:**

small B-cell neoplasms can show plasmacytic differentiation and may potentially progress to aggressive lymphoma (DLBCL). Epstein-Barr virus (EBV) infection may cause the transformation of malignant cells *in vitro*.

**Design and Method:**

we established VR09 cell line with plasmacytic differentiation, obtained from a case of atypical, non-CLL B-cell chronic lymphoproliferative disease with plasmacytic features. We used flow cytometry, immunohistochemistry, polymerase chain reaction, cytogenetic analysis and florescence *in situ* hybridization in the attempt at thoroughly characterizing the cell line. We showed VR09 tumorigenic potential *in vivo*, leading to the development of activated DLBCL with plasmacytic features.

**Results:**

VR09 cells displayed plasmacytic appearance and grew as spherical tumors when inoculated subcutaneously into immunodeficient Rag2^−/−^ γ-chain^−/−^ mice. VR09 cell line and tumors displayed the phenotype of activated stage of B cell maturation, with secretory differentiation (CD19+ CD20+ CD79a+ CD79b+/− CD138+ cyclin D1- Ki67 80% IgM+ IgD+ MUM1+ MNDA+ CD10- CD22+ CD23+ CD43+ K+, λ- Bcl2+ Bcl6-) and they presented episomal EBV genome, chromosome 12 trisomy, lack of c-MYC rearrangement and Myd88 gene mutation, presence of somatic hypermutation in the VH region, and wild-type p53.

**Conclusion:**

This new EBV-positive cell line may be useful to further characterize *in vivo* activated DLBCL with plasmacytic features.

## Introduction

Human lymphoma cell lines and animal models have contributed significantly to better understand the physiopathology of hematopoietic tumors. Cell lines are generally characterized by monoclonal origin, differentiation arrest and sustained proliferation *in vitro*, while maintaining most cellular features and specific genetic alterations of the primitive tumor [1]. However, primitive malignant cells may change some morphological and phenotypic features in culture, due to Epstein-Barr virus (EBV) infection. EBV is a B lymphotropic herpesvirus linked to several B cell malignancies and capable of transforming human B cell into permanent lymphoblastoid cell line *in vitro* [2]. Besides Hodgkin lymphoma, Burkitt lymphoma, human immunodeficiency virus-associated lymphomas and post-transplant lymphoproliferative disorders [3], EBV has been also detected in low-grade lymphoproliferative diseases such as chronic lymphocytic leukemia (CLL) where it has been shown to correlate with transformation to Richter syndrome in some cases [4], [5], [6].

Many types of small B lymphoid neoplasms can demonstrate plasmacytic differentiation and specific immunophenotypic or genetic markers for plasmacytic lymphoma; furthermore, small B-cell lymphomas with plasmacytic differentiation frequently represent a diagnostic challenge, and sometimes only a diagnosis of small B-cell lymphoma with plasmacytic differentiation can be done [7]. All of these disorders may potentially undergo transformation to large-cell lymphoma [8].

Diffuse large B-cell lymphoma (DLBCL) represents a very heterogeneous group of aggressive lymphomas. Following the introduction of gene expression profiling (GEP), it has become increasingly clear that DLBCL is a biologically and clinically heterogeneous disorder. Two major categories are now recognized, i.e. germinal center B-cell (GCB) and activated B-cell (ABC) types, which are different for the expression of more than 1000 genes. In the attempt at traslating GEP classification into daily practice, several immunohistochemical algorithms have been proposed as a surrogate for GEP analysis [9].

Some aggressive DLBCL show the phenotype of terminal B-cell differentiation and represent a continuum spectrum of lesions that ranges from conventional activated DLBCL to plasma cell disorders with specific molecular patterns [10], some of which, including Myd88, have been recently suggested to correlate with plasmacytic lymphoma histotype [11], [12].

We describe here the established VR09 cell line, an EBV-positive cell line with plasmacytic differentiation displaying tumorigenic potential *in vivo* and leading to the development of activated DLBCL with plasmacytic features when injected into Rag2^−/−^ γ-chain^−/−^ mice. Thus, VR09 xenotrasplantation can be used successfully as preclinical model for human DLBCL with plasmacytic differentiation to further characterize this disease.

## Materials and Methods

### Cell Collection and Culture

**Figure 1:**
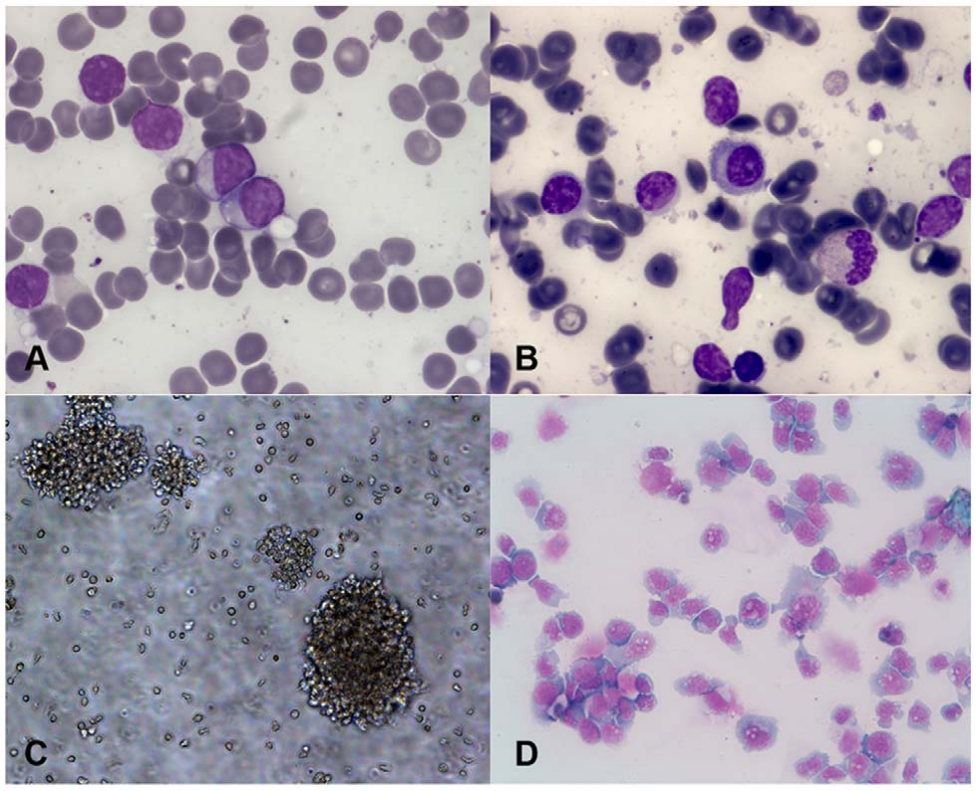
Morphology of primary malignant cells and VR09 cell line. Morphological features of patient’s primary cells and VR09 cells in suspension, as assessed by May-Grünwald-Giemsa staining. (A) Patient’s peripheral blood cells. (B) Patient’s bone marrow cells. (C) VR09 cells: small and round clumps in suspension. (D) VR09 cells: plasmacytoid appearance, with irregular nucleus, compact chromatin and abundant basophilic cytoplasm.

A 75-year old Caucasian man was admitted to hospital in September 2008 for fever, neutropenia and lymphocytosis (WBC 20.1×10^9^/L, with neutrophils 0.8×10^9^/L and lymphocytes 18.2×10^9^/L), and moderate anemia and trombocytopenia (Hb 9 g/dl, PLTS 93×10^9^/L). No significant superficial lymphoadenopathy or splenomegaly were present. Peripheral blood smear shows the predominance of small-medium sized mature lymphoid cells with abundant cytoplasm and compact chromatin **(** **Figure 1A** **)**. A bone marrow sample was sent to our laboratory for first level-immunophenotyping by flow cytometry (FACSCanto, Becton Dickinson Biosciences, CA, USA), after written informed consent, as approved by the Ethics Committee of Azienda Ospedaliera Universitaria Integrata Verona (N. Prog. 1828, May 12, 2010 - ‘Institution of cell and tissue collection for biomedical research in Onco-Hematology’). Bone marrow smear appeared infiltrated (60%) by a cell population of lymphoplasmocytoid elements of small-medium size (**Figure 1B**). First level-immunophenotyping showed the presence of a cell population expressing CD19, CD20, CD22, CD138, surface immunoglobulins (sIg) at high level, and negative for CD5, CD10, ZAP-70, thus suggesting the diagnosis of non-CLL atypical B-cell chronic lymphoproliferative disease with plasmacytic features. No other exams could be performed, as the patient died of sepsis the following day.

Mononuclear cells were purified from bone marrow sample by Ficoll-Paque centrifugation (Lymphoprep, Fresenius Kabi Norge AS for Axis-Shield Poc AS, Oslo, Norway), washed in phosphate-buffered saline solution (PBS) and resuspended at 1×10^6^/mL concentration in RPMI 1640+ GlutaMAX 1X containing 10% heat-inactivated fetal bovine serum and 1% penicillin/streptomycin (all from GIBCO, Invitrogen). Cells were cultured in 75 cm^2^ flask and incubated in humidified 5% CO_2_ atmosphere at 37°C. Half of culture medium was replaced every 3–4 days maintaining the same cell density of 1×10^6^ cells/mL. To determine growth kinetics, cells were seeded at lower density (350,000/mL) and counted at 0, 24, 48 and 72 hours by flow-cytometry (FACSCanto, Becton Dickinson, Italy). No mitogens or growth factors were added during culture. Cells were maintained in culture up to one year. Cell morphology was evaluated on cytospins stained with May-Grunwald Giemsa dye.

### Immunophenotypic Analysis

Cell vitality was assessed by acridine-orange/ethidium bromide staining and epifluorescent microscopy. Aliquots of 3×10^5^ cells were incubated for 15 minutes at room temperature with three-color combinations of appropriate monoclonal antibodies anti-human CD3, CD19, CD20, CD22, CD23, CD25, CD38, CD43, CD45, FMC-7, CD79b 7AAD (Becton Dickinson, Italy), CD5, CD10, K, λ, IgG, IgM, IgD (Dako, Italy), CD103 (Beckman Coulter, Italy), CD138 (Cytognos, Italy), and isotype controls (Becton Dickinson, Italy). Samples were analyzed by FacsCANTO flow cytometer with BD FACSDiva software (Becton Dickinson, Italy).

### Cell Cycle

For determination of the DNA content, 1.5×10^5^ cells were incubated with 1 ml of staining solution including 5 ml of hypotonic solution, 50 µg of propidium iodide (Bender MedSystems) and 20 µg of RNAse for two hours at 4°C analyzed using appropriate settings by FacsCanto flow cytometer. Human peripheral mononuclear cells were used as control for the comparison of S fraction.

### DNA and RNA Extraction, cDNA Synthesis

DNA and RNA were obtained from 10^7^ cells by AllPrep DNA/RNA/Protein Mini Kit (Quiagen, Hilden, Germany). DNA quality was verified by spectrophotometry and RNA quality by the Agilent Bionanalyzer 2100; 1 µg of total RNA was reverse-transcribed by using SuperScript III First-Strand Synthesis System (Invitrogen, Carlsbad, California) and cDNA was used as a template for reverse transcriptase polymerase chain reaction (RT-PCR) amplification using listed in **Table 1**.

**Table 1:** Primers used to amplify and sequence P53, Card 11, CD79B, MYD88 AND RPMS1 genes.

Primers	Sequence	Ta°	Amplicon lenght
P53-1-F	AAGTCTAGAGCCACCGTCCA	55°C	771 bp
P53-1-R	AAGTGTTTCTGTCATCCAAATACTC		
P53-2-F	AGCCAAGTCTGTGACTTGCA	55°C	851 bp
P53-2-R	GGGGAACAAGAAGTGGAGAA		
Card11-1-F	AGATGCAACGGGAGCCTGGC	55°C	631 pb
Card11-1-R	AGGTTAGCAGCTCCACGCGC		
Card11-2-F	GGCCAAGGACCTGCAACGCT	55°C	703 pb
Card11-2-R	CCGCTCCACCTCCTCCAGCT		
Card11-3-F	GAGGCCCTGGAGGACAGGCA	55°C	690 pb
Card11-3-R	TCCGCAGGAGCTAGGGCTGG		
Card11-4-F	TCCTGCCCTACCATCCGCCC	55°C	671 pb
Card11-4-R	CAGCAGCTGGTGGCCCTCAC		
Card11-5-F	TCCCAGCTCACCCTGCTGGG	55°C	739 pb
Card11-5-R	CCGAGATGATGCGGACCCGC		
Card11-6-F	CCCGTCTCTCGCGAGCAAGC	55°C	928 pb
Card11-6-R	CGTCTGCTGGGGCAGCTCTG		
CD79B-1-F	GCCTCGGACGTTGTCACGGG	55°C	858 pb*
CD79B-1-R	TGGGCCAGCTTCAGAGGCCA		
MYD88-EX1F	CGCAGGAGAAAGAGGAAGC	60°C	491 bp
MYD88-EX1R	ATGGGAGACAGGATGCTGAG		
MYD88-EX3F	TGGGTAAAGAGGTAGGCACTCCCAG	60°C	275 bp
MYD88-EX2R	GCCCATCTGCTTCAAACACCCATGC		
MYD88-EX3F	AAGCCTTCCCATGGAGCTCTGACCAC	60°C	311 bp
MYD88-EX3R	GCTAGGAGGAGATGCCCAGTATCTG		
MYD88-EX3F	ACTAAGTTGCCACAGGACCTGCAGC	60°C	194 bp
MYD88-EX3R	ATCCAGAGGCCCCACCTACACATTC		
MYD88-EX3F	GTTGTTAACCCTGGGGTTGAAG	60°C	297 bp
MYD88-EX3R	GCAGAAGTACATGGACAGGCAGACAGATAC		
RPMS1F	GGATGGGAGAGGGTGATCTT	60°C	151 bp
RPMS1R	ACGTGGAGTTTGCAGTCCTC		

Isoform 1: 858 pb; Isoform 2: 546 pb; Isoform 3: 861 pb.

### Analysis of VH Rearrangement, p53, CD79B, Card 11 and MYD88 Mutation

To verify the identity between cells from the patient and the cell line, rearranged *VH* genes were analyzed as previously described [13] in frozen DNA from both the patient and the cell line *TP53, CD79B* and *CARD11* genes were amplified and sequenced by using the primers reported in **Table 1**. *MYD88* exons 1 to 5 were sequenced as previously described [11], [12]. VH and CD79B PCR bands were excised from agarose gels and purified by using spin columns (PureLink Quick Gel Extraction and PCR Purification Combo Kit, Invitrogen). PCR products were sequenced by dye terminator reaction (Big Dye Terminator Cycle Sequencing Kit v.3.1, Applied Biosystems, Warrington, UK) on AB3130XL automated sequencer (Applied Biosystems). *TP53*, *CARD11* and *MYD88* PCR products were directly purified with magnetic beads (Agencourt AMPure XP, Beckman Coulter Genomics, Beverly, Massachussets, USA) and sequenced as reported above and by outsourcing to Base Clear BV (Leiden, The Netherlands).

VH genes sequences were compared to published germline sequences using IgBLAST (http://www.ncbi.nlm.nih.gov/igblast/). Sequences with 2% or less deviation from any germline IgVH sequence were considered unmutated. *TP53, CD79B*, *CARD11* and *MYD88* amplified sequences were compared to reference sequences by using Geneious software (Biomatters Ltd., Auckland, New Zealand).

### Preparation of Formalin-fixed Paraffin-embedded (FFPE) Cell Block and Immunohistochemical Staining

Briefly, 12×10^6^ cells were centrifuged, washed once in PBS and then incubated for 15 minutes in 4% buffered formalin. Cells were centrifuged again and resuspended in 1 ml of 1% low melting point agarose at 37°C; then, they were poured into disposable plastic moulds and cooled for ten minutes at +4°C. Solid agarose blocks were put inside a histological cassette, with 4% buffered formalin at room temperature, and then processed routinely as tissue block; 4 µm-thick sections were cut from the block and stained with ematoxilin-eosin to verify the quality of the cell inclusion. Many sections from the block were immunolabeled with the broad panel of antibodies described in **Table 2**. A 25% cut-off was used to distinguish positive from negative. All samples were processed by using a sensitive ‘Bond polymer Refine’ detection system in automated Bond immunostainer (BondMax, Vision-Biosystem, Menarini, Florence, Italy).

**Table 2:** Marker expression by VR09 cell line and tumor masses, as assessed by immunohistochemistry performed on FFPE cell block.

Antibody	Species	Clone	Source	Diluition	VR09 cell line	Tumor masses
Annexin a1	mouse	B01P	Abnova	100	neg	neg
Bcl-2	mouse	124	Dako	40	pos	pos
Bcl-6	mouse	LN22	Novocastra	20	neg	neg
Cyclin D1	rabbit	SP4	Neomarkers	10	neg	neg
CD03	rabbit	SP7	Labvision	150	neg	neg
CD05	mouse	4C7	Novocastra	200	neg	neg
CD10	mouse	56C6	Novocastra	50	neg	neg
CD19	mouse	LE-CD19	Serotec	200	pos	pos
CD20	mouse	L26	Novocastra	100	pos	pos
CD22	mouse	FPC1	Novocastra	20	pos	pos
CD23	mouse	1B12	Novocastra	100	pos	pos
CD25 IL2R	mouse	4C9	Novocastra	100	pos/neg	pos/neg
CD38	mouse	38C03	Neomarkers	50	neg	pos/neg
CD43 (T cell)	mouse	MT1	Novocastra	30	pos	pos
CD79alfa	mouse	JCB117	Dako	100	pos	pos
CD79beta	mouse	JS01	Novocastra	50	pos/neg	pos/neg
CD123	mouse	7G3	Bd	100	neg	pos/neg
CD138 (syndecan-1)	mouse	BB4	Serotec	50	pos/neg	pos/neg
DBA44 hairy cell	mouse	DBA.44	Dako	20	neg	neg
FOXP1	mouse	JC12	Abcam	500	neg	pos/neg
EBV (EBER)		mRNA probe*	Vision Byosistems		pos	pos
GCET1	mouse	RAM341	Abcam	500	neg	neg
HHV-8	mouse	13B10	Novocastra	50	neg	neg
IgD	rabbit	polyclonal	Dako	20	pos	pos/neg
IgG	rabbit	polyclonal	Dako	10000	neg	neg
IgM	rabbit	polyclonal	Dako	5000	pos/neg	pos/neg
Ki-67	mouse	MM1	Novocastra	20000(15′ incubation)	pos(40%)	pos(80%)
ZAP-70	mouse	2F3.2	Upstate	200	neg	neg
κ chains	rabbit	polyclonal	Dako	50	pos	pos
λ chains	rabbit		Dako	30000	neg	neg
MNDA	mouse	235	provided by CNIO**	2	pos	pos
MUM1 Protein	mouse	MUM1p	Dako	50	pos	pos
PAX 5	mouse	1EW	Novocastra	50	pos/neg	pos/neg
P53 Protein	mouse	DO-7	Novocastra	20	pos/neg	pos/neg
Sox11	rabbit	polyclonal	Atlas Sigma	50	pos/neg	pos/neg
TCL1A	mouse	1–21	Santa Cruz	100	pos/neg	pos/neg
TdT	rabbit	polyclonal	Dako	30	neg	neg
TRAcP	mouse	26E5	Neomarkers	30	neg	neg

### Establishment of the Mouse in vivo Model

Three month-old immunodeficient Rag2^−/−^ γ-chain^−/−^ mice (Taconic animal models, New York’s River Valley, NY USA) were injected either subcutaneously (s.c.) (n = 6) with 5×10^6^ VR09 cells, resuspended in 0.2 ml PBS, or intravenously (i.v) (n = 6) with 0.5×10^6^ VR09 cells resuspended in 0.1 ml PBS. Animals were checked three times a week for the development of tumors. When s.c. tumors developed up to approximately 2 cm of diameter, corresponding to a spheric volume of approximately 4.187 cm^3^, mice were killed. Half of the tumor mass was frozen and half included in paraffin according to standard procedures. Sections of 5–8 µm from each tumoral mass were prepared and analyzed for morphology, immunohistochemistry and FISH. Paraffin sections for immunohistochemistry were stained with a panel of antibodies, as described above (**Table 2**). Moreover, a little portion of the mass from two of six s.c injected mice, was dissected and mechanically dissociated into single-cell suspension; 1.5×10^6^ cells from each tumor were cultured in RPMI +10% FBS and incubated as described above. Immunophenotipic analysis was performed on secondary cell culture after two months of culture, as previously described.

Three of six mice injected i.v. were euthanized at +30 days, while the others were sacrificed after four months from the treatment. Spleen, liver, femurs, lymph nodes, lungs and bowel of the i.v. injected animals were collected and included in paraffine. Tissue sections were immunostained with antibodies against CD20 and CD138 to verify the presence on tumor infiltration. In addition, immunophenotyping with anti-human CD19 and CD45 monoclonal antibodies and isotype controls was performed by standard procedures on peripheral blood mononuclear cells of all mice injected both s.c. and i.v.

This study was carried out in strict accordance with the recommendations in the Guide for the Care and Use of Laboratory Animals of the National Institutes of Health. The *in vivo* studies were approved by Verona University Ethical Committee for experimentation on animals (Prot. n°51 del 16/06/11, D.lgs 116/92).

### EBV Status Evaluation

The presence of EBV was assessed on both cells cultured *in vitro* and cells obtained from the tumoral mass developing after *in vivo* injection of VR09 cell line. Epstein-Barr virus-encoded RNA (EBER) hybridization was performed on cytoinclusion by means of specific fluorescein-conjugated EBER probes (Bond ISH EBER Probe, Vision-Biosystem, Menarini, Florence, Italy). EBER sequence was detected by anti-fluorescin antibody associated with a sensitive ‘Bond polymer Refine’ chromogenic detection system in an automated immunostainer (Bondmax, Vision-Biosystem, Menarini, Florence, Italy).

To verify that EBV infection of VR09 derived directly from the patient, the presence of EBV gene RPMS1 was detected by PCR amplification on both DNA from the patient and VR09 cell line. The reaction was performed in a final volume of 50 ml, containing 5 ml Taq Buffer Advanced (5 Prime, Hamburg, Germany), 5 pmol of each primer (listed in Table 1), 200 mM each dNTP and 1.25 U of Taq DNA Polymerase (5 Prime). Cycling conditions were 2 minutes at 94°C, 20 seconds at 60°C, 40 seconds at 68°C, then a final elongation of 5 minutes at 68°C. PCR products were analyzed on a 2100 Bioanalyzer (Agilent Technologies, Waldbronn, Germany), using the Agilent DNA1000 chip and reagents. Electronic data were analyzed using the manufacturer’s software (Agilent 2100 Expert).

### Karyotyping and Fluorescence in situ Hybridization (FISH)

Cytogenetic analysis was performed with standard methods on both cells cultured *in vitro* and cell suspensions from tumors developing after *in vivo* injection of VR09 cell line: 10–20×10^6^ cells were centrifuged, resuspended in RPMI 1640 medium containing 20% fetal bovine serum (FBS, Lonza, Verviers, Belgium) and cultured for 24 hours. Before harvest, 0.15 µg/ml colchicin (Eur-Clone, Italy) was added. Chromosome preparation and staining using QFQ technique was performed according to standard protocols. Karyotypes were scored according to the International System for Human Cytogenetic Nomenclature (ISCN) [14]. Images were captured with a ZEISS Axioplan microscope (ZEISS, Jena, Germany) and evaluated by Cytovision applied imaging system (Molecular Devices, New Milton).

Interphase cytogenetic FISH was performed on nuclei from VR09 cell suspension and formalin-fixed and paraffin-embedded tissue sections from *in vivo* tumor masses developing after *in vivo* injection of VR09 cell line. The following kits were used: LSI BCL-2 dual color probes for 14q32;18q21 (Abbott-Vysis, Olympus), LSI CCND1 (11q13) break-apart probes (Abbott-Vysis, Olympus), LSI IGH/CCND1 probe for 11q13-14q32.3 (Abbott-Vysis, Olympus), LSI MYC dual color break-apart probe for 8q24 (Abbott-Vysis, Olympus), LSI C-MYC dual color probes for 8q24-14q32 (Abbott-Vysis, Olympus) and CEP (centromeric) mapping 12p11.1-q11.1 (Abbott-Vysis, Olympus). Chromosome 9 probes from human painting box kit (Spectral Imaging) were also used to identify whole chromosome 9 on metaphases of cell derived from *in vivo* tumor masses. The procedure was performed according to the methods described elsewhere [15].

FISH slides were examined by using either Axioplan (Zeiss, Germany) or Olympus BX61 (Olympus, Hamburg, Germany) epifluorescent automated microscopes. The signals were recorded using a CCD camera (Axiocam HRm, Zeiss and Digital Camera Olympus), and score was assigned according to manufacturer’s instructions, available in each commercial FISH kit.

## Results

### Establishment and Characterization of VR09 Cell Line

Primary cells proliferated very slowly during the first six weeks of culture, with a consequent decline in cell number. Remaining cells formed a few clusters of proliferation and started growing in suspension forming small round clumps (**Figure 1C**). At ten weeks of culture a stable proliferating cell line (designed VR09) was established, with a doubling time of approximately 84 hours. May-Grünwald-Giemsa staining showed a wide cellular size spectrum, from medium to large cells, with plasmacytoid features and occasional bizarre shapes: cells had a round-ovoid, often eccentric or moderately irregular nucleus, with compact chromatin and abundant basophilic cytoplasm (**Figure 1D**). At present, VR09 cells grow in suspension in RPMI 1640 medium supplemented with 10% heat-inactivated FBS without requiring any other supplements, and can be vitally stored in a medium consisting of 60% RPMI 1640 medium, 30% heat-inactivated FBS and 10% DMSO. Once thawed and put in culture, VR09 cell line grows rapidly and maintains the same doubling time of the early culture. Cells are optimally maintained at 500,000 and 1,000,000/mL cell density and may be split 1∶5 every 72 hours.

### Immunophenotype and Immunohistochemistry

**Figure 2:**
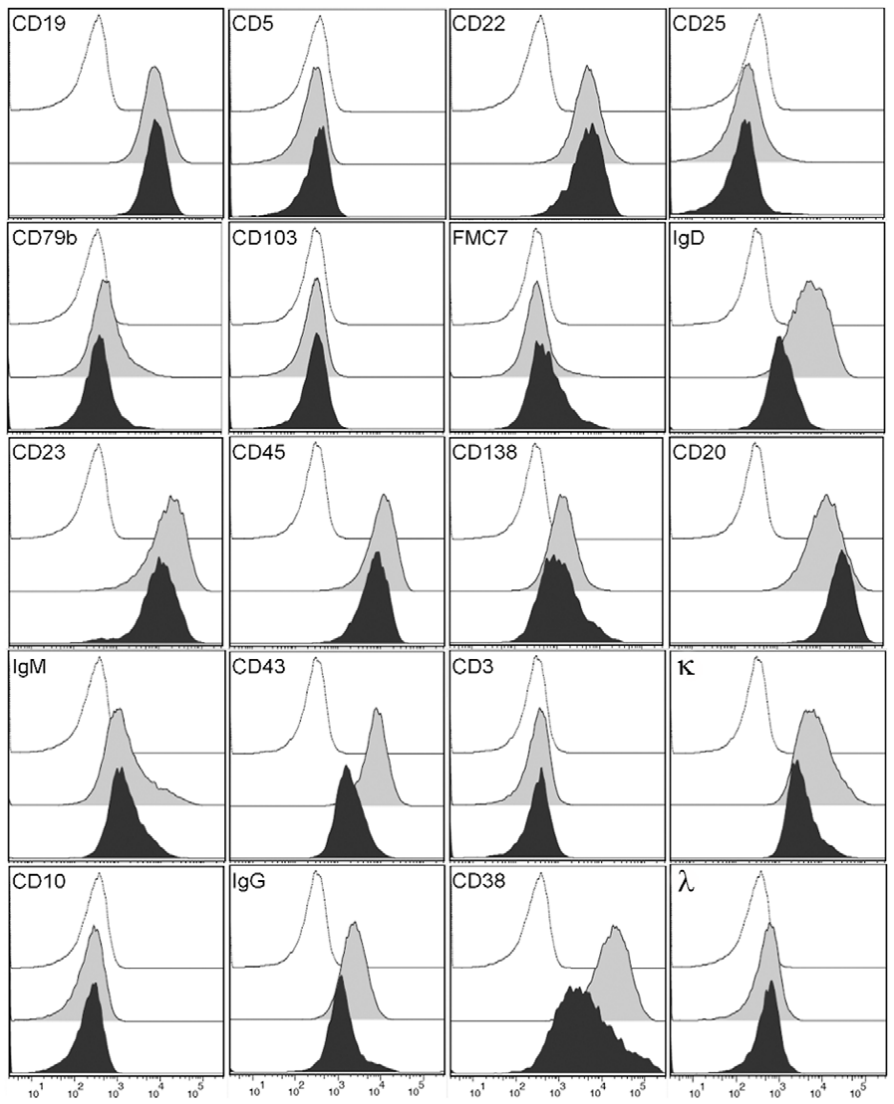
Immunophenotype. Immunophenotyping of VR09 cell line (grey and filled curves) and cell suspensions from *in vivo* tumor mass (black and filled curves), as compared with isotype control (white curves).

Immunophenotyping by flow cytometry was performed on VR09 cell line at 10 weeks and one year after continuous culture, resembling the phenotype of atypical B-cell chronic lymphoproliferative disease with plasmacytic features found in the patient. In particular, cells were CD45^+^, CD19^+^, CD20^+^, CD22^+^, CD23^+^, CD43^+^, CD38^+^, CD138^+^, IgD^+^, IgM^+^, IgG^+^ and kappa chains^+^, and negative for CD3, CD5, CD10, CD25, CD79b, CD103, FMC7 and lambda chains (**Figure 2**); Zap-70, as expected, was not expressed. VR09 surface markers remained unchanged over time, with the exception of CD20, whose expression had a 1 log increase as compared to starting culture.

Results of immunohistochemistry performed on FFPE cell block are summarized in **Table 2**: all the markers shown by flow cytometry were confirmed, with the exception of IgG and CD38 expression that resulted negative by immunohistochemistry; proliferation index (Ki-67+ cells) was about 40%. In addition, cells were positive for Bcl-2, MNDA [16] and MUM1, and negative for Bcl-6, Cyclin D1, Annexin A1, DBA44, GCET1, ZAP-70, TdT and TRAP; other markers, such as PAX-5, Sox11, FOXP1 and TCL1, were weakly and variably expressed. In summary, neoplastic cells displayed a late B-cell phenotype (MNDA+, FOXP1+, IRF4/MUM1+, cyK+, CD138+), while germinal center markers (Bcl-6, CD10, TCL1, GCET1) and immature B-cell markers (TdT) were negative. Cyclin D1 was included to exclude the remote possibility of a blastoid variant of mantle cell lymphoma, and also because plasma cell myeloma can be cyclin D1-positive. Hairy cell leukemia markers (Annexin A1, DBA44, TRAP) were investigated to exclude the very remote possibility of evolution from hairy cell leukemia (in consideration of the clinical setting).

### Cell Cycle

DNA content of VR09 cell line was analyzed at six months and one year of culture. High S-phase rate was observed (diploid S 22.66%), in absence of tetraploid or aneuploid peak, as compared to the control (diploid S: 1.64%).

### IVGH, TP53, CARD11, CD79B and MYD88 Mutation Analysis

VR09 cell line and the DNA from the patient showed the same VH3-7/D4-23/JH4 gene rearrangement. The comparison of the sequence with published germline sequences showed 95.7% identity in both VR09 cell line and DNA from the patient, with evidence of somatic hypermutation (SHM) in the VH region, defined as more than 2% mutations as compared to germline sequences. The analysis of the sequences obtained from both strands of cDNA and compared with the published *TP53* sequence (U94788.1) showed that this sequence in VR09 cell line was wild-type *(data not shown)*. *CARD11* sequence was compared to the published one (NM_032415): VR09 cell line showed a silent heterozygous mutation leading to D533D variation in the protein linker region. Finally, VR09 cell line expressed all the three isoforms of the CD79B gene (NM_000626.2; NM_021602.2; NM_001039933.1): cDNA sequence analysis showed that variants 1 and 3 had an homozygous silent mutation in exon 3 leading to C122C variation in the Ig-like V-type protein domain, involving the disulfide bond; moreover, variant 2 had an Alanine insertion in the exon 2b corresponding to the protein signal peptide region. The analysis of exons 1 to 5 of *MYD88* showed no mutations compared to the reference sequence (NG_016964).

### Mouse Model

**Figure 3:**
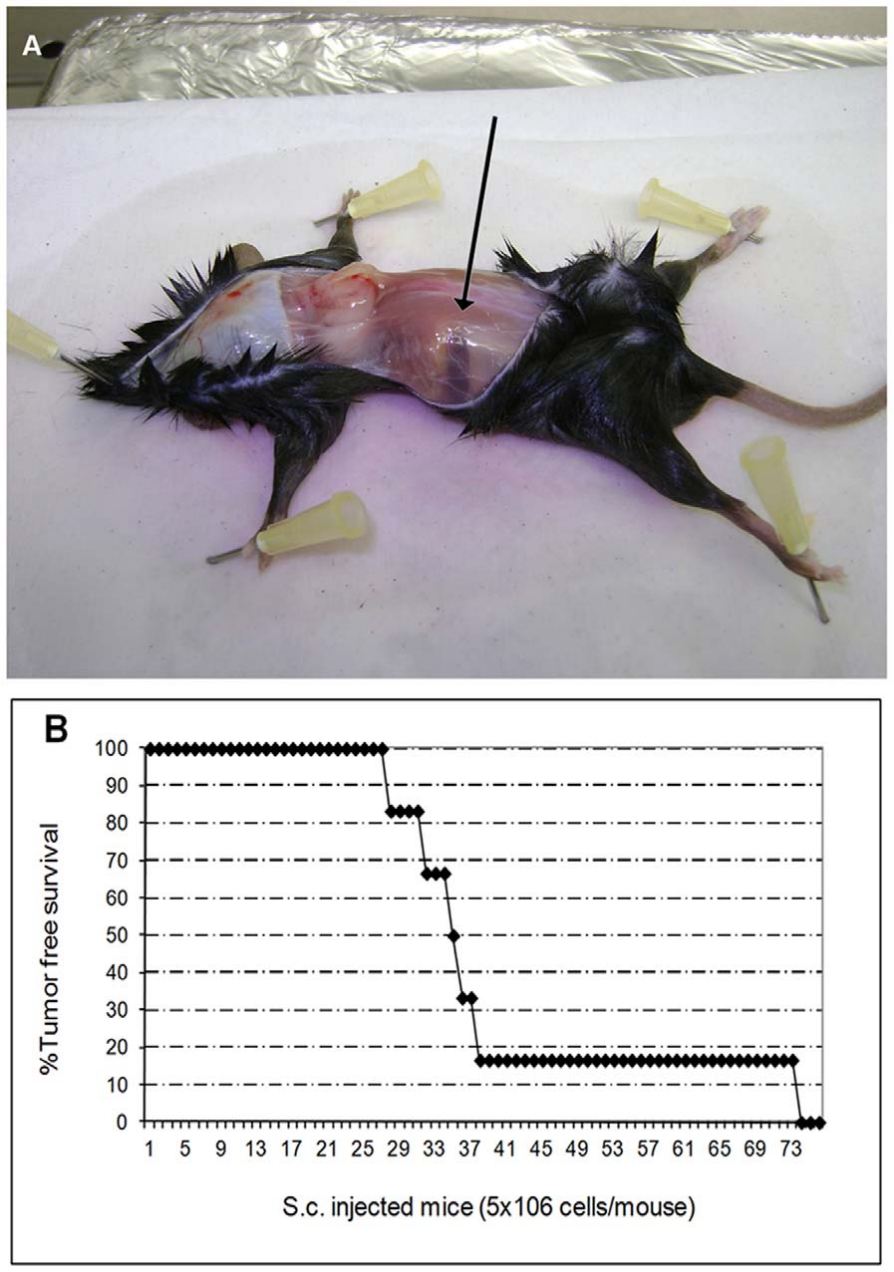
Cell line growth in vivo. ( A) Spherical subcutaneous mass (arrow) in Rag-2^−/−^ γ-chain^−/−^ mice 36 days after VR09 cell line injection (representative case). (B) Timing of tumor development *in vivo* (6 mice s.c. injected with 5×10^6^ cells/mouse).

**Figure 4:**
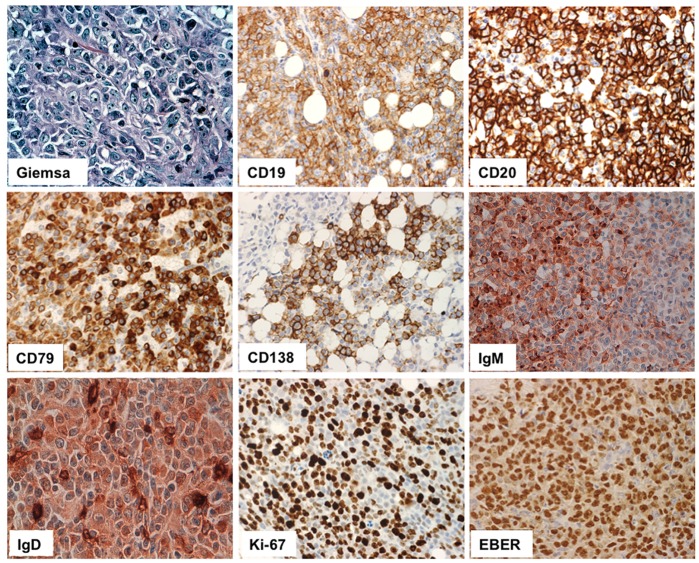
Representative histological markers detected on tumors. High magnification (20X) of histological sections of tumors developing after *in vivo* injection of VR09 cell line. Lymphoid infiltrates display large size, plasmablastic-plasmacytic features and high Ki-67 index. Cells express CD19, CD20, CD138, CD79a, IgM, IgG and EBV protein (EBER).

All six mice injected s.c. with VR09 cell line developed spherical tumor masses around the site of injection by 34–74 days after treatment and were sacrificed 10 days later. Subcutaneous mass and tumor incidence *in vivo* are shown in **Figure 3**. Cells from subcutaneous masses were evaluated by H&E and Giemsa staining: cells ranged from medium to large size, with irregular nuclei, condensed chromatin without nucleoli and basophilic cytoplasm. Immunohistochemistry showed no significant differences in tumor cell immunophenotype as compared to VR09 cell line in culture (**Table 2** and **Figure 4**).

Cells suspensions obtained by disaggregation of *in vivo* tumor masses showed proliferative capacity when cultured with the same procedure used for primary VR09 cell line. Again, cells appeared as small and round clumps with plasmacytoid features and bizarre shape: immunophenotyping on these cells after two months of continuous secondary culture did not show any significant change of cell surface marker expression as compared to primary VR09 cell line (**Figure 2**).

Of the six i.v. injected mice, three were euthanized after 30 days from injection and the other 3 after four months: histopathologic examination of spleen, liver, femurs, lymph nodes and lungs did not show any evidence of tumor infiltration. In addition, no circulating human CD45/CD19-positive cells were detected in peripheral blood of both s.c. and i.v. injected mice.

### EBV Status

EBV RNA evaluation was performed on FFPE cell blocks and tumoral masses by using a fluorescein-conjugated probe coupled to chromogenic detection (Bond ISH EBER Probe, Vision-Biosystem, Menarini, Florence, Italy). In situ hybridization for EBV-encoded RNA (EBER) showed a clear nuclear signal in both VR09 cell line and tumors (**Table 2** and **Figure 4**).

**Figure 5:**
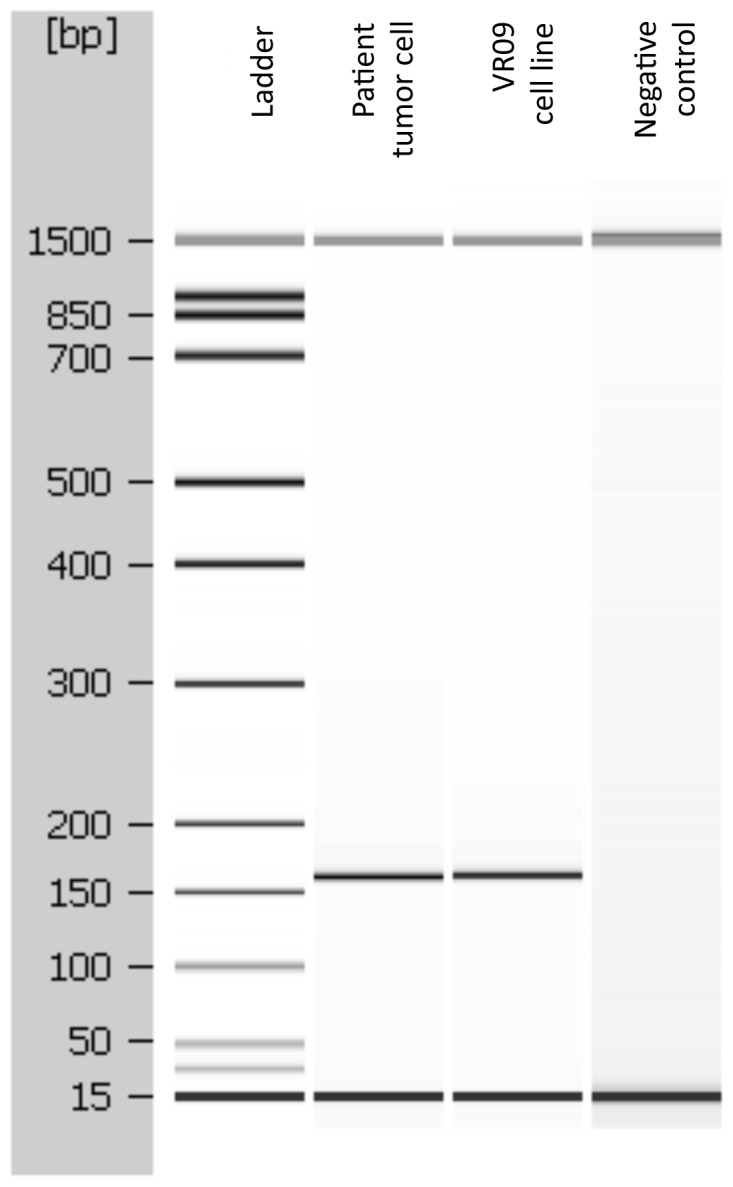
EBV positivity in the original tumor and in the VR09 cell line. PCR products analysis by Agilent 2100 Bioanalyzer showed the presence of the same 151 bp specific amplicon for EBV RPMS1 gene, thus demonstrating that EBV infection was present in the original cells from patient. A normal DNA from pancreas was used as negative control.

Analysis of the RPMS1 gene PCR products, performed on both DNA from patient and VR09 cell line, showed the same 151 bp specific amplicon (**Figure 5**), thus showing that EBV infection was already present at the beginning of culture.

### Karyotyping

**Figure 6:**
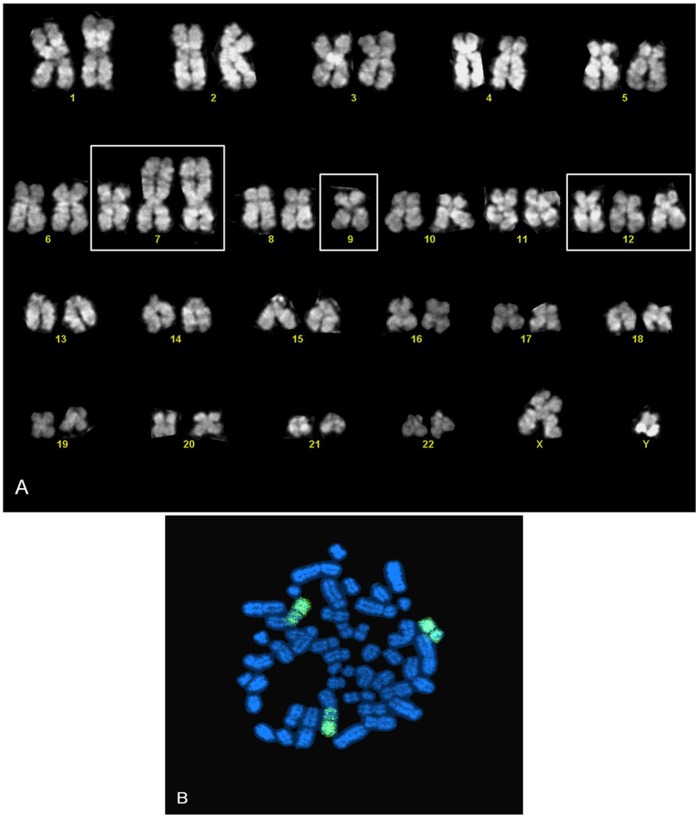
Karyotyping and FISH of secondary culture. (A) Trisomy of chromosome 12 and structural chromosome aberration involving chromosomes 7 and 9 on secondary culture detected by karyotype; (B) Chromosome 9 staining by FISH of secondary culture.

Chromosome analysis performed at 10 weeks and one year of continuous culture showed a male karyotype with 47 chromosomes. All metaphases exhibited trisomy of chromosome 12. Karyotype was repeated on secondary cell cultures obtained by disaggregation of *in vivo* tumor masses, thus revealing the presence of additional structural chromosome aberration involving chromosomes 7 and 9, i.e. 47, XY, der(7)(9qter->9p23::7p13->7qter), +der(7)(9qter->9p23::7p13->7qter), −9, +12 (**Figure 6A**).

### Fluorescence in situ Hybridization (FISH)

Adjacent or fused fluorescent signals (LSI BCL-2) were found in 5% of nuclei on tissue sections; this percentage is below the established 10% signal threshold for considering a case positive (adjacent signals are generated by random overlapping of genomic regions). No break-apart fluorescent signals were found for LSI CCND1 (11q13) and LSI MYC (8q) probes on tissue sections. No adjacent or fused fluorescent signals were found for LSI IGH/CCND and LSI C-MYC dual color probes (4% of nuclei, below threshold). Trisomy of chromosome 12 was found in 45% of nuclei. The rearrangement involving chromosomes 9 and 7 detected by karyotyping on cell suspension from *in vivo* tumor mass was confirmed by fluorescent signals derived from hybridization of chromosome 9 DNA probes to target chromosome 9 (chromosome 9 paint) (**Figure 6B**). The main features of VR09 cell line are summarized in **Table 3**.

**Table 3:** Summary of VR09 cell line features.

Parameter	Features
***Cell Line***	
Name of cell line	VR09
Cell line type	Lymphoid
Cell phenotype	CD19+ CD20+ CD79a+ CD79b+/− CD138+ cyclin D1- Ki67 80% IgM+ IgD+ MUM1+ MNDA+ CD10− CD22+ CD23+ CD43+ K+, λ− Bcl2+ Bcl6−
Cytogenetic karyotype	Trisomy of chromosome 12
Mutation analysis	VH3-7/D4-23/JH4
Tumorigenic capacity	Subcutaneously growth into immunodeficient Rag2^−/−^ γ-chain^−/−^ mice
***Clinical data***	
Primary disease of patient	Atypical B-cell chronic lymphoproliferative disease with plasmacytic features
Disease Status	At diagnosis
Patient data (age, race, sex)	75-year old Caucasian man
Source of material	Bone marrow
Year of establishment	2008
***Cell culture data***	
Culture Medium	RPMI 10% FBS
Subcultivation routine	Maintained at 0.5–1×1.0^6^ cells/mL; 1∶5 split every 72 hours
Minimum cell density	0.5×10^6^ cells/mL
Maximum cell density	1.0×10^6^ cells/mL
Doubling time	84 hours
Cell storage condition	60% RPMI 1640 medium, 30% heat-inactivated FBS and 10% DMSO
In situ morphology	medium-to-large sized cells, plasmacytoid features and occasional bizarre shapes; round clumps in suspension
Viral status	EBV-positive

## Discussion

We describe here the establishment and molecular characterization of novel human EBV-positive DLBCL cell line with plasmacytic differentiation obtained from a patient with atypical B-cell chronic lymphoproliferative disease with plasmacytic features.

Human B-lymphoid cell lines are extensively used world wide as models in studies of various aspects of B cell biology and as a tools in research on the pathogenesis of leukemia and lymphoma. Nevertheless, the efficiency establishment of new leukemia and lymphoma cell line is rather low and remains a random process [1]. Sometimes, the spontaneous proliferation and immortalization of normal or malignant cells *in vitro* is due to EBV infection. It is well known for several decades that infection of normal B cell with EBV leads to establishment of lymphoblastoid cell line, thus confirming its oncogenic potential [17]. EBV is associated to several malignancies *in vivo* and may lead to spontaneous growth and transformation of malignant cells *in vitro* [2]; nevertheless, lymphoproliferative cell lines from CLL-like chronic lymphoproliferative disorders have never been reported, unless infected by EBV or in prolymphocytoid transformation [18]. The establishment of VR09 cell line was likely due to EBV infection; in fact, as EBV was already present in patient’s B cells, we can reasonably assume that the infection drove the transformation into DLBCL once cultured *in vitro* and injected into mice, thus excluding a culture-dependent contamination by EBV. Indeed, the Association between EBV infection and indolent B-cell neoplasms, such as chronic lymphocytic leukemia, marginal zone lymphoma, lymphoplasmacytic lymphoma has been reported [4], [19], [20] Thus, the positivity of EBV in VR09 cell line is not surprising. Moreover, some studies showed that cases of DLBCL developed from low-grade lymphoproliferative diseases are EBV-positive and that EBV may have a potential role, although not yet completed defined, in the progression of the indolent disease [21], [22].

Unfortunately, we lack much information about the patient; however, bone marrow cell morphology and immunophenotype are in agreement with the diagnosis of atypical, non-CLL B-cell chronic lymphoproliferative disease with plasmacytic features. Low-grade B-cell lymphoproliferative disorders, such as CLL/small lymphocytic lymphoma and marginal zone B-cell lymphoma may have overlapping features, thus making the differential diagnosis sometimes difficult. Furthermore, many types of small B lymphoid neoplasms can display plasmacytic differentiation and a phenotype resembling lymphoplasmacytic lymphoma; the latter consists of small lymphocytes, plasmacytoid lymphocytes and plasma cells expressing IgM and pan-B-cell antigens, such as CD19, CD20 and CD22, and usually negative for CD5, CD10 and CD23. As we lack information about serum IgM paraprotein, we concluded as a case of atypical B-chronic lymphoproliferative disease with plasmacytic differentiation, rather than a plasmacytic lymphoma.

Among lymphoproliferative disorders, VR09 cell line presents features of DLBCL with plasmacytic differentiation, with medium to large sized and plasmablastic/plasmacytic-like cells, high Ki-67 index and activated phenotype according to Hans’ and new immunostaining algorithm [9]. Moreover, cells are positive for MUM1, CD38, and CD138, thus suggesting a terminal-B cell differentiation.

Although lymphoplasmacytic lymphoma and other lymphoproliferative disease with plasmacytic differentiation may develop into diffuse large B cell lymphoma, similarly to CLL, the frequency of transformation is low [22]. Consequently, VR09 cell line can be useful as a model of DLBCL variant corresponding to a very late or activated stage of B cell maturation [23], i.e. the early post-germinal stage differentiation, not entirely terminally differentiated.

Despite the aggressive transformation of indolent disease is not always clonally related to original disease, some data are indirectly consistent with the hypothesis of clonal evolution: in fact, VR09 cell line maintains the immunophenotype and plasmacytoid features of the primary cells at diagnosis, displays the presence of the same clonal gene rearrangement found in patient’s DNA and has a tumorigenic capacity *in vivo*; the latter feature strongly suggests its malignant behaviour. VR09 cell line grow subcutaneously without dissemination in peripheral blood and no engraftment when it is administrated i.v. This strange peculiarity could be simply due to the *in vivo* model used, i.e. Rag2^−/−^ γ-chain^−/−^ mice. They are immunodeficient mice with C57B16/10 background devoid of functional B and T cells because of the complete lack of function of the V(D)J recombinase enzyme system, and displaying deficient innate immunity due to the absence of γ chain of interleukin-2-receptor [24]. This model has the advantage to show a stable phenotype without developing spontaneous tumors, as compared to SCID mice [25]. Indeed, VR09 cell line injection led to 100% incidence of developing tumors *in vivo*, always maintaining the same features.

We found trisomy of chromosome 12 in our model. This aberration is often detected in chronic lymphocytic leukemia [26], [27] and in other chronic B cell malignancies, including sometimes lymphoplasmacytic lymphoma. We also found an additional chromosomal abnormality involving chromosomes 7 and 9 after secondary culture: genetic alterations of cell lines are usually stable over the time, but it is debated whether cell line may acquire additional rearrangements in culture [1].

We also found a synonymous variant of CD79B involving the insertion of Alanine in the exon 2b of variant 2. This variant may be physiological since it has been detected in other isoforms; however, it has never been described before. Moreover, a recent study suggested that also synonymous variants may be involved in the pathogenesis of some diseases [28]; therefore, one could speculate that this novel variant of CD79B gene could be involved in the pathogenesis of lymphomas. VR09 cell line did not display the common missense mutations of Card11, usually involved in the constitutive activation of NF-kB pathway in some cases of human activated-B-cell like DLBCL [29]. However, the detection of synonymous mutations of Card11 reveals the accuracy of sequencing method. Interestingly, VR09 cell line showed unmutated Myd88 gene, differently from what described in recents reports [11], [12], thus suggesting that lymphomas with plasmacytic features may have different gene patterns.

In conclusion, VR09 is a new cell line of activated DLBCL with plasmacytic differentiation that grow as solitary tumor once injected s.c. in immunodeficient mice. This model could be useful for further studies about the development of DLBCL in patients with low-grade B-cell lymphoproliferative disorders with plasmacytic differentiation, which is a rare but possible event in clinical practice.
